# Genomic ecology of Marine Group II, the most common marine planktonic Archaea across the surface ocean

**DOI:** 10.1002/mbo3.852

**Published:** 2019-07-02

**Authors:** Olivier Pereira, Corentin Hochart, Jean Christophe Auguet, Didier Debroas, Pierre E. Galand

**Affiliations:** ^1^ Sorbonne Université, CNRS, Laboratoire d'Ecogéochimie des Environnements Benthiques (LECOB), Observatoire Océanologique de Banyuls Banyuls sur Mer France; ^2^ Laboratoire Microorganismes: Génome et Environnement, UMR 6023 CNRS – Université Blaise Pascal Aubière France; ^3^ Marine Biodiversity, Exploitation and Conservation (MARBEC) Université de Montpellier, CNRS, IFREMER Montpellier France

**Keywords:** 16S rRNA, *Euryarchaeota*, global ocean, metagenomics, poseidoniales, proteorhodopsin

## Abstract

Planktonic *Archaea* have been detected in all the world's oceans and are found from surface waters to the deep sea. The two most common *Archaea* phyla are *Thaumarchaeota* and *Euryarchaeota. Euryarchaeota* are generally more common in surface waters, but very little is known about their ecology and their potential metabolisms. In this study, we explore the genomic ecology of the Marine Group II (MGII), the main marine planktonic *Euryarchaeota,* and test if it is composed of different ecologically relevant units. We re‐analyzed *Tara* Oceans metagenomes from the photic layer and the deep ocean by annotating sequences against a custom MGII database and by mapping gene co‐occurrences. Our data provide a global view of the distribution of *Euryarchaeota*, and more specifically of MGII subgroups, and reveal their association to a number of gene‐coding sequences. In particular, we show that MGII proteorhodopsins were detected in both the surface and the deep chlorophyll maximum layer and that different clusters of these light harvesting proteins were present. Our approach helped describing the set of genes found together with specific MGII subgroups. We could thus define genomic environments that could theoretically describe ecologically meaningful units and the ecological niche that they occupy.

## 1 INTRODUCTION

The pioneering works published Fuhrman, McCallum, and Davis (1992) and DeLong (1992) revealed the presence of aerobic and mesophilic archaea in both costal surface waters and in the deep ocean. Today, planktonic archaea are known from nearly every marine environment and they are classified in four major phylogenetic groups (see Santoro et al. (2019) for a recent review). Among these groups, the Marine Group II (MGII) (DeLong, 1992) was defined within the phylum *Euryarchaeota* originally delineated by Woese, Kandler, and Wheelis (1990). A recent study based on metagenome‐assembled genomes (MAGs) now proposes MGII as an order‐level lineage that would be named *Candidatus* Poseidoniales (Rinke et al., 2018).

On the basis of the 16S rRNA gene sequences, MGII has been dived into two main monophyletic groups, the MGIIa and the MGIIb (Massana, DeLong, & Pedrós‐Alió, 2000). MGIIa is composed mostly of surface microorganisms, while MGIIb contains principally taxa found below 200 m depth, although it can also be detected in the upper water column (Deschamps, Zivanovic, Moreira, Rodriguez‐Valera, & López‐García, 2014; Galand, Casamayor, Kirchman, Potvin, & Lovejoy, 2009; Martin‐Cuadrado et al., 2015; Massana et al., 2000). MGIIa also dominated the polar ocean archaeal community (Galand et al., 2009), while in the northwestern Mediterranean Sea, MGIIa and MGIIb showed different seasonal dynamics; MGIIa being predominant in summer, when the nutrients become depleted, and MGIIb in winter when nutrients are more abundant (Galand, Gutiérrez‐Provecho, Massana, Gasol, & Casamayor, 2010; Hugoni et al., 2013). In addition, a MGIIa genome was assembled from surface ocean metagenomes (Iverson et al., 2012) and genomic fragments from a MGIIb with a low GC content was recently reconstructed from deep chlorophyll maximum samples and proposed as representative for a new class called *Thalassoarchaea* (Martin‐Cuadrado et al., 2015). These observations draw the picture of a complex ecological structure within the MGIIa and MGIIb, and the question remains as whether the phylogenetic diversity observed within the clades corresponds to the presence of different ecologically relevant taxa.

Rinke et al. (2018) now propose that the newly named order *Candidatus* Poseidoniales (formerly MGII) should be divided in two families: *Candidatus* Poseidonaceae fam. nov. (formerly subgroup MGIIa) and *Candidatus* Thalassarchaeaceae fam. nov. (formerly subgroup MGIIb) (Rinke et al., 2018). Within these families, the authors resolved 21 genera, named by letters, and many had distinct geographic distributions and metabolisms. Recently, another MAG based study resolved 17 distinct subclades (Tully, 2019) and thus confirmed the existence of most of the genera defined by Rinke et al. (2018).

There is to date no cultured MGII representatives and their lifestyle is thus not well known. Information from metagenomics, and reconstructed or partially assembled genomes allow, however, a glimpse into some potential metabolisms (Rinke et al., 2018; Tully, 2019). The light‐harvesting capability of some MGII living in the photic zone was first deduced from the presence of genomics fragments that encoded a proteorhodopsin protein (Frigaard, Martinez, Mincer, & DeLong, 2006). This proteorhodopsin could support a photoheterotrophic lifestyle by generating a light‐driven chemiosmotic potential (Frigaard et al., 2006; Iverson et al., 2012). Summer peaks of abundance of MGIIa in the surface waters of the Mediterranean Sea could thus be associated to photoheterotrophy (Hugoni et al., 2013). Recently, proteorhodopsin baring *Euryarchaeota* were separated in two clades: one with a blue light signature typical for deep photic waters and the other with a green light signature in shallow photic waters (Rinke et al., 2018). Another study separated five different subclades of MGII proteorhodopsin (Tully, 2019) following nomenclature from (Boeuf, Audic, Brillet‐Guéguen, Caron, & Jeanthon, 2015; Pinhassi, DeLong, Béjà, González, & Pedrós‐Alió, 2016). Other metagenomics fragments suggest that some MGIIa could live associated to particles, and that they could be motile and degrade polymers like proteins and lipids (Iverson et al., 2012; Rinke et al., 2018; Tully, 2019; Xie et al., 2018). Recently, Xie et al. (2018) presented a new partially reconstructed MGIIa genome, the MGIIa_P, which contained higher proportions of glycoside hydrolases indicative of the ability to hydrolyse glycosidic bonds. In addition, and for the first time, a catalase gene was identified. Catalases could protect against oxygen species generated by the abundant phototrophs present in the eutrophic Pearl River Estuary (Xie et al., 2018). Recent MAG reconstructions confirmed the presence of glycoside hydrolases in MGIIa representatives suggesting that they are degrading algal substrates (Rinke et al., 2018; Tully, 2019). Algal particle scavenging MGIIa also have the potential for motility or adhesion via an archaeal flagellum‐based system (Li, He, Yan, Chen, & Dai, 2017; Rinke et al., 2018; Tully, 2019). For MGIIb, several genomic features support the idea of aerobic heterotrophic metabolisms based on different substrates like proteins, carbohydrates, fatty acids, and lipids (Li et al., 2017; Rinke et al., 2018; Tully, 2019). Genes for amino acid, transcript of amino acid transporter, carbohydrate, and lipid transport have also been identified (Baker et al., 2013; Deschamps et al., 2014; Rinke et al., 2018; Tully, 2019). In addition, genes affiliated to sulfate reduction were found in deep sea MGII (Martin‐Cuadrado et al., 2008, 2015; Moreira, Rodríguez‐Valera, & López‐García, 2004), which suggests anerobic respiration under low‐oxygen conditions (Orsi et al., 2015). The recent report of nitrate reductase genes in *Candidatus* Thalassarchaeaceae fam. nov. (MGIIb) is an additional indication of the ability of some MGIIb to adapt to ecosystems with low oxygen availability (Rinke et al., 2018).

The main goal of this work was first to explore at a global scale and at different ocean depths the distribution of MGII *Euryarchaeota*. We further aimed at identifying subgroups within the MGIIa and MGIIb and verify if they could be associated to specific environmental conditions and specific functional genes. To do so we analyzed 135 metagenomic samples collected during the 2.5 year *Tara* Oceans circumnavigation (Sunagawa et al., 2015). We grouped MGII 16S rRNA gene sequences according to their K‐mer signature and tested the distribution of these groups against environmental parameters. We then calculated patterns of co‐occurrences between 16S rRNA and functional genes to infer the genomic environment of MGII subgroups.

## 2 MATERIAL AND METHODS

### 2.1 Metagenomic data

We focused on 135 *Tara* Oceans metagenomes corresponding to 63 stations that comprised 63 samples from the surface ocean (SRF), 42 from the deep chlorophyll maximum (DCM), and 30 from the mesopelagic zone (MES) (Table S1). We targeted the free living bacterial size fraction for all these samples. The metagenomes were sequenced using the Illumina technology as described earlier (Sunagawa et al., 2015).

All the sequence analysis methods used in this study are summarized in Figure S1. For the taxonomic assignation, we annotated the 16S rRNA gene sequences (miTAGs) available on the *Tara* Oceans companion web site (http://ocean-microbiome.embl.de/companion.html) at a higher resolution than previous work. For the functional genes, we downloaded all the metagenome reads available on the EBI website (www.ebi.ac.uk/ena/data/view/PRJEB402). We also downloaded the assemblies from the 135 metagenomes (www.ebi.ac.uk/ena/about/tara-oceans-assemblies). In addition, we used the Ocean Microbial Reference Gene Catalog (OM‐RGC) of 40,154,822 genes from the *Tara* Oceans companion web site (Sunagawa et al., 2015).

### 2.2 Marine group II clusters, 16S RRNA database and annotation

To classify the Marine Group II *Euryarchaeota*, we constructed a new database based on 7,645 (length >900 nt) Marine group II (MGII) sequences from SILVA 128 (Quast et al., 2013). The MGII SILVA sequences were grouped into clusters according to their K‐mer content using VizBin (Laczny et al., 2015) and the clusters were separated by a multidimensional reduction (VizBin software) (Laczny et al., 2015). Multiple iterations were performed and optimal results were determined by counting penta‐mers (parameters: PCA method = mtj; theta = 0.5; perplexity = 100). The MGII sequences separated into 35 different 16S rRNA gene clusters (File S1). We then identified the clusters annotated as MGIIa, MGIIb by BLASTn against reference sequences. In a second step, we re‐annotated the SILVA database following our cluster affiliation (cluster1 to cluster35) and obtained a reference database composed of 608,679 unique 16S rRNA gene sequences including 690 full length MGII sequences.

The 14,090,466 *Tara* Oceans 16S rRNA gene sequences were annotated against our database by blastn with standard parameters (Altschul et al., 1997). An abundance table was constructed by grouping together sequences according to nucleotide identity cutoffs (Yarza et al., 2014): >75% identity for the phylum level and >97% identity for the genus level.

### 2.3 Co‐occurrence network between gene‐coding sequences and MGII clusters

Gene‐coding sequences were obtained by mapping the 17,039,492,256 high‐quality *Tara* Oceans reads (7,699,091,255 for SRF, 5,060,990,056 for DCM and 4,279,410,945 for MES) against the Ocean Microbial Reference Gene Catalog using the BWA‐MEM aligner with the options: bwa mem ‐M ‐t 10. The mapped reads were filtered using a minimum mapping quality of 10 and sequences were counted to form an abundance matrix. The 500,000 most‐abundant gene‐coding sequences in the abundance matrix were used for co‐occurrence analyses after normalization per million of reads.

The co‐occurrence between the relative abundance of MGII clusters and the relative abundance of all gene‐coding sequences was obtained by calculating a Maximum Information Coefficient measure (MIC) using the MINE software (Reshef et al., 2011). The software also computes a Pearson correlation used to determine positive and negative relationships. The positive correlations (Pearson r >0) with the strongest co‐occurrences (MIC >0.9) were used to build networks with weighted spring embedded layout in Cystoscape (Shannon et al., 2003).

The gene‐coding sequences identified in the network were then taxonomically annotated using top BLASTn result against the nonredundant nt database (release 2018‐06‐08) containing 48,103,425 nucleotides sequences. A functional annotation was done with BLASTx (E value <10^−5^) against the KEGG (Kanehisa & Goto, 2000) and the UniRef90 databases (UniProt Consortium, 2018).

Genes coding for the proteorhodopsin was extracted from the dataset and compared by BLASTx against reference sequences for the pop, pop1, pop2, pop3, and pop4 clusters defined earlier (Iverson et al., 2012).

Colored KEGG pathway maps were produced with MetaPath Explorer (v0.1.1) (Hochart & Debroas ) or each MGII clusters from the list of KEGG Orthology ID identified within the gene‐coding sequences.

### 2.4 Gene conservation in contigs

*Tara* Oceans contigs containing MGII 16S rRNA gene sequences were identified by BLASTn against our 16S rRNA database. A total of 450 contigs were affiliated to MGII and we further analyzed the largest contigs. A total of five MGII contigs with more than 23,000 nt were annotated with the Rapid Annotation tool on the RAST platform (http://rast.nmpdr.org). The comparison of the contigs was done with EasyFig (Sullivan, Petty, & Beatson, 2011).

### 2.5 Statistics

Relations between the relative abundance of MGII clusters and noncollinear explanatory geomorphologic variables (depth, longitude, and latitude) and physicochemical variables (temperature, salinity, oxygen, nitrate, nitrite, phosphate, nitrite/nitrate, silicate, PAR) were examined by redundancy analysis. Prior to analyses, collinearities in the environmental variables were tested. Variables with collinearity up to 0.85 according to Spearman correlations were grouped together, and proxies of each group were used as explanatory variables. All explanatory variables were standardized in order to avoid scale effect in subsequent multivariate analysis and multiple stepwise regressions. The explanatory variables were obtained after a stepwise model selection using the ordistep function of the vegan package in R. Partial RDA (pRDA) was used to remove variability effects due to explanatory variable not included in the model, and the remaining variability was assumed to be due to depth, temperature, oxygen, nitrates, nitrite, phosphate, latitude.

A SIMPER test was performed with the vegan package in R to identify the MGII clusters that contributed the most to the difference between water layers.

Richness was calculated with phyloseq package in R and a *t* test was used to test significant differences between the depth layer.

## 3 RESULTS

### 3.1 Construction of the MGII 16S rRNA clusters according to their K‐mer signature

The MGII 16S rRNA gene sequences were separated into 35 clusters according to their K‐mer content (Figure S2). The clusters contained different numbers of SILVA reference sequences ranging from 1 to 64 (Figure S3). Five clusters contained more than 50 sequences (clusters 5, 6, 15, 21, 25). Seven clusters had fewer than five sequences (clusters 17, 22, 24, 26, 27, 28, 35).

**Figure 1:**
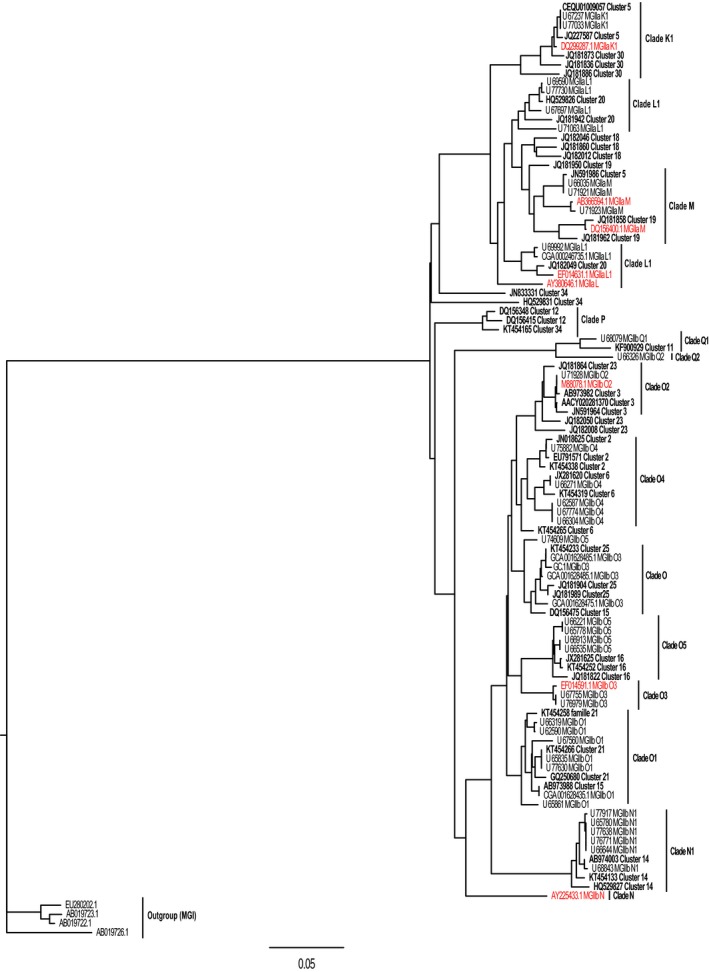
Phylogenetic tree calculated using SSU rRNA sequences from this study K‐mer clusters (in bold) and reference sequences from Rinke et al., 2018 and Galand et al., 2010 (in red). Distances were calculated with the Kimura2 algorithm and the tree computed with FITCH in Phylip

The use of K‐mer separated clearly sequences earlier annotated as MGIIa and MGIIb and grouped according to clades defined earlier from the Mediterranean (Galand et al., 2010) and the recent genera defined by Rinke et al. (2018) (Figure 1). The tree pinpoints positions on the phylogenetic tree where earlier genome data are not sufficient and where new clades can still be discovered. Sequences affiliated to the newly defined *Thalassoarchaea mediterranei* and *Thalassoarchaea marina* were separated into different MGII clusters.

### 3.2 Global distribution of archaea

**Figure 2:**
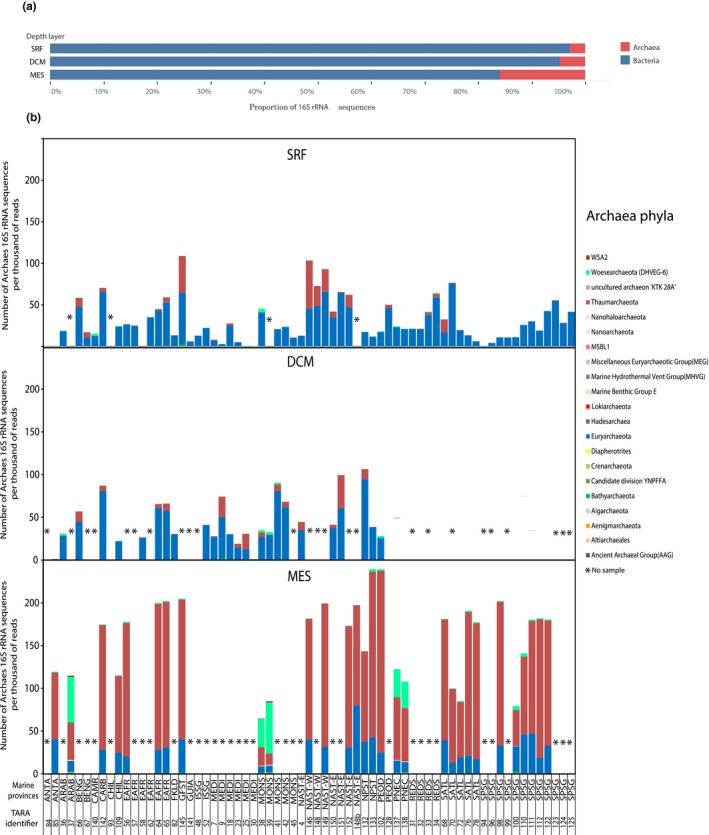
Proportion of Archaea and Bacteria 16S rRNA sequences in surface (SRF), deep chlorophyll maximum (DCM), and mesopelagic zone (MES) of the global ocean (a). Number of 16S rRNA sequences belonging to different archaeal phyla in *Tara* Oceans samples (b). Sequence counts are normalized per thousand reads. Samples are grouped according to marine provinces see Table S1 for marine provinces abbreviations. *No sample available

The proportion of archaea at the domain and phylum level was calculated after annotating 16S rRNA gene sequences extracted from the TARA metagenomes (Figure S1). We detected 892,510 archaea sequences in the *Tara* Oceans dataset, among which 367,179 were MGII sequences. Overall, in the global ocean the proportion of archaea increased with depth (Figure 2a). Archaea were more abundant in the deep mesopelagic zone (MES) where they represented on average 16% of the sequences. At the deep chlorophyll maximum (DCM) they represented 4.8% of the prokaryotic sequences and at the surface (SRF) only 2.9% (Figure 2a).

Archaea were ubiquitous in the *Tara* Oceans samples, with the exception of TARA_085 (surface layer, Polar Ocean) where no archaea 16S rRNA gene sequences were detected (Figure 2b). The number of sequences annotated as archaea varied greatly from close to 0–108 archaea per thousand 16S rRNA gene sequences in the SRF layer and from 0 to 164 in the DCM. In the MES all stations contained archaeal 16S rRNA gene sequences and their number varied between 64 and 239 per thousand.

At the phylum level, we found four different patterns: (a) surface waters where *Euryarchaeota* dominated the archaea 16S rRNA gene sequences, (b) some surface and DCM samples where both *Euryarchaeota* and *Thaumarchaeota* were abundant, (c) the mesopelagic zone where *Thaumarchaeota* dominated, (d) some samples with many sequences affiliated to the phylum *Woesearchaeota*. *Woesearchaeota* sequences were found in five different MES stations, three from the Indian Ocean (TARA_037, 038 and 039), and one from the north Pacific Ocean (TARA_138). In the DCM, *Woesearchaeota* were abundant in TARA_137 where they represented 1/3 of the archaea sequences. We also identified representatives of less common phyla such as the *Bathyarchaeota* or the newly described *Woesearchaeota* phylum present in some deep‐sea samples from the India Ocean and from north Pacific Ocean in large proportions (Figure 2).

### 3.3 Marine group II community composition

**Figure 3:**
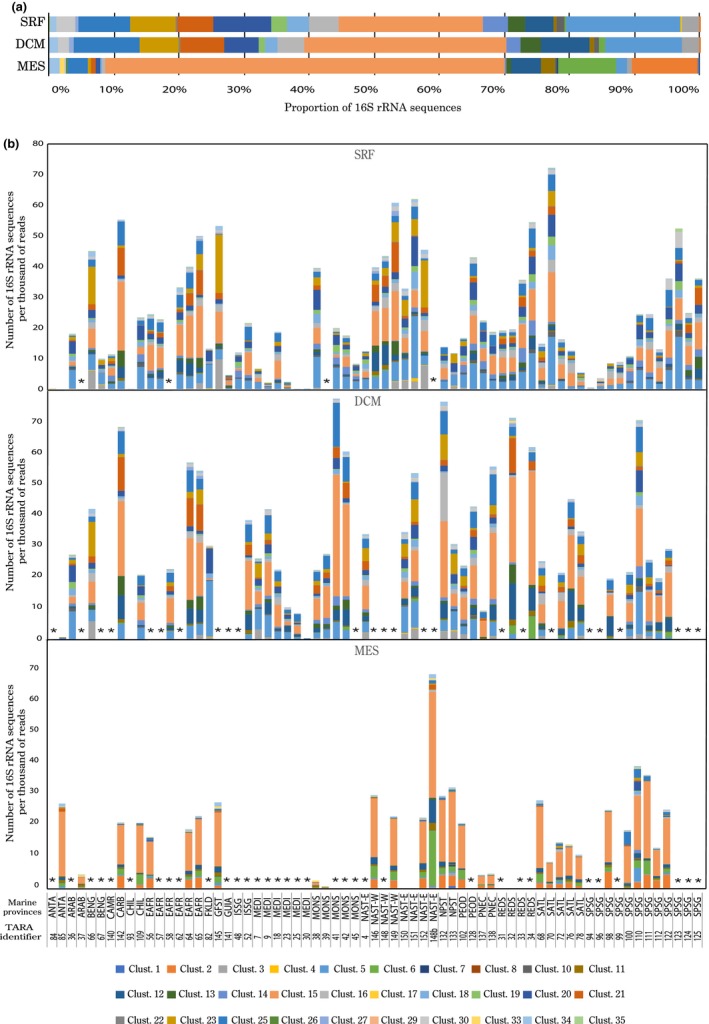
Average proportion of the different MGII clusters in the surface (SRF), deep chlorophyll maximum (DCM), and mesopelagic zone (MES) of the global ocean (a) and in all the *Tara* Oceans samples (b)

At the cluster level, we performed a SIMPER test to identify the cluster that contributed the most to the difference between each water layer among the 135 *Tara* Oceans samples (Figure 3). Overall, clusters 2, 15, and 6 were typical for the mesopelagic zone (MES) where their proportion reached respectively 10.2%, 61.5%, and 9.1% of the total MGII 16S rRNA gene sequences versus 0%, 22.1%, and 1.1%, respectively, in the surface. Clusters 5, 25, 20 were more abundance in the SRF and the DCM layers (Figure 3a) (17.6%, 7.9%, 8.9% in SRF and 11.7%, 10.2%, 5.5% in DCM vs. 1.7%, 3.2%, 0.7% in MES). Difference between SRF and DCM were less marked, but cluster 20 and 23 were more present in the SRF and cluster 12, 21 were more abundant in the DCM.

### 3.4 Relation between MGII cluster distribution and environmental parameters

**Figure 4:**
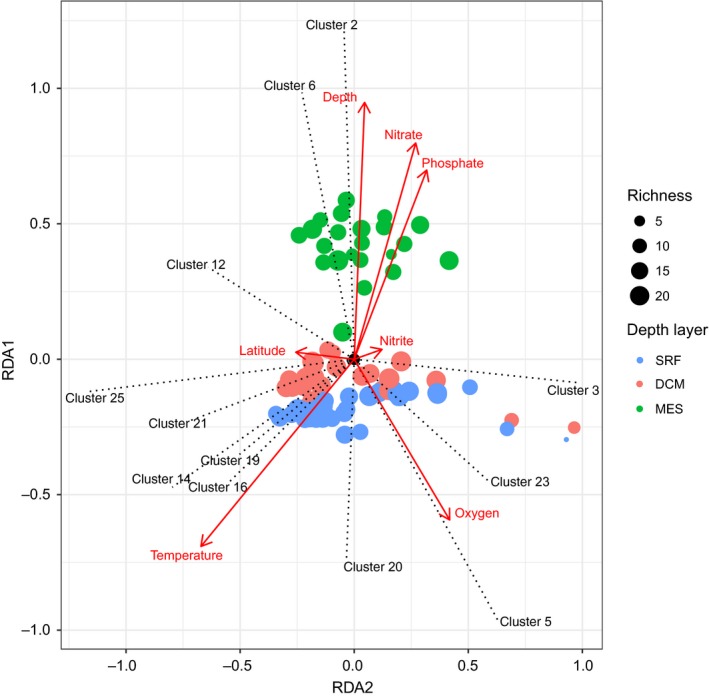
Ordination plot of the partial Redundancy Analysis (pRDA) ran on the MGII cluster abundance matrix. Physicochemical parameters were included as co‐variables. Arrows represent the parameters that explain the ordination. Dotted lines show the main clusters contributors. Colors represent depth layers: surface (SRF), deep chlorophyll maximum (DCM), and mesopelagic zone (MES)

The pRDA analysis showed that MGII communities were separated in three groups along axis 1 (Figure 4). The first group was composed of MES samples, the second contained DCM samples, and the last contained SRF samples. SRF and DCM samples were more dispersed along axis 2. Seven significant environmental variables explained 52% of the total variance information. Most of this variance (44.5%) was associated to the first axis, which was correlated to depth, nitrate, and phosphate (0.94 for depth, 0.79 for nitrate, and 0.69 for phosphate) (Figure 4). Temperature and oxygen correlated with the second axis (0.67 for temperature and 0.41 for oxygen).

We further observed that two groups of clusters correlated with axis 1: cluster 2 and cluster 6, which were in the same direction as depth, and cluster 20 in the opposite direction (Figure 4). For axis 2, we found two clusters with high correlation in opposite direction: cluster 3 and cluster 25, but without correlation to environmental variables. Temperature pointed in the same direction as cluster 16, and oxygen toward cluster 5.

MGII cluster Richness was significantly higher in the mesopelagic zone than in the DCM (*t* test, *p* < 0.01).

### 3.5 Co‐occurrence of MGII clusters and functional genes

**Figure 5:**
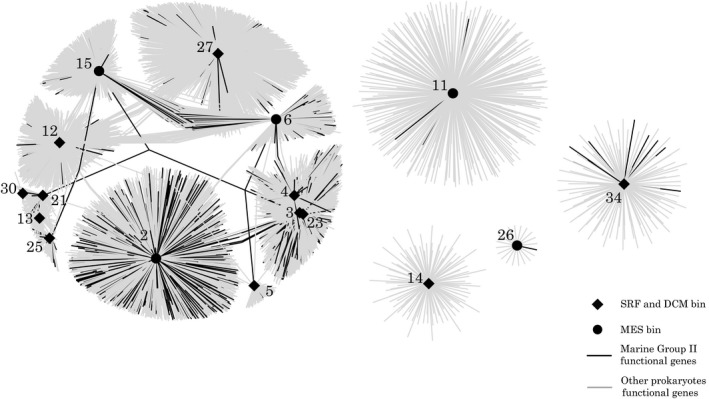
Network visualization showing co‐occurrences between MGII clusters and gene‐coding sequences from the global ocean. Co‐occurrences were determined by the maximal information coefficient (MIC). The black diamonds represent clusters typical of the surface and the deep chlorophyll maximum layers and the black circles show the clusters typical of the mesopelagic zone. The black lines represent connections to gene‐coding sequences taxonomically annotated as MGII and the gray lines represent connections to other prokaryotes

We constructed a co‐occurrence network by computing MIC correlations between the MGII 16S RDNA genes and all archaeal and bacterial functional genes of the dataset. The co‐occurrence analysis revealed 25 MGII clusters that were highly correlated to 10,661 genes. The network represents clusters that had more than 11 connections to genes (Figure 5). A group of 13 clusters grouped together because they were associated to common genes. The four other clusters were isolated from each other and each was associated to a specific set of genes (Figure 5). The number of cluster associated genes varied and ranged from 2,443 for cluster 2 (Table S2) to 20 for cluster 26. A BLAST analysis against the nr database (E value <1^−10^, bit score >250) revealed that 17% of the genes (*n* = 1,819) had a taxonomic affiliation. Among them, 13% of the annotated genes (*n* = 1,462) were identified as archaea (Table S2). Among archaea, 41% were affiliated to the *Euryarchaeota* and 35% to MGII. Most of the identified MGII genes were associated to the cluster 2 (Figure 5).

A functional annotation showed that 12.9% of the genes were associated to a KEGG pathway. For example, we identified sequences coding for all the subunits of the ABC branched‐chain amino acid transporter in cluster 11, all subunits for ABC lipopolysaccharide transporters in cluster 15. Other clusters were associated to genes covering only incomplete protein complex like for fragmented iron ABC transporter complex (clusters 2, 11, 12, 14, 23, 27) or ferric transport system *Aflu*A and/or *Aflu*B and/or *Aflu*C subunits (clusters 2, 11, 12, 14, 23, 27) (Figure S4).

We also detected sequences coding for enzyme involved in sulfur metabolism pathways, particularly in cluster 2 and 12, which were associated to sequences coding for the transformation of taurine to sulfite and taurine‐clustering periplasmic protein (*Tau*A). Clusters 2 and 4 were associated to assimilatory sulfate reduction genes while cluster 14 co‐occurred with the dissimilatory sulfate reduction and oxidation gene *Apr*ABA. The *Sox* gene was associate to cluster 12 (Figure S5).

Enzymes involved in fatty‐acid and/or amino‐acid metabolisms were also identified. Cluster 27 had most associations to almost complete amino acids biosynthesis pathways, including the synthesis of methionine, glycine, valine, leucine, isoleucine, lysine, arginine, threonine, glutamine, and proline (Figure S6). Cluster 2 had genes for the biosynthesis of Valine, Leucine, Glycine, Isoleucine, Threonine, and Glutamine. A total of 14 clusters co‐occurred with genes for cytoplasmic (*n* = 3) and/or mitochondrial (*n* = 11) fatty‐acid metabolisms. In addition, several genes coding for fatty acid degradation pathway were identified. The pathways were not complete, but a large portion was founded in clusters 27 and 12 (Figure S7). Cluster 12 had genes coding for the transformation of long chain fatty acid to long chain acyl‐[acyl‐carrier‐protein] and for fatty‐acid liberation and CoA.

The BLASTp annotation against the UniRef90 database increased the proportion of annotated genes to 81% (*n* = 8,601) (Table S3). Close to 35% (*n* = 3,010) of them showed archaeal functions, among which 82% (*n* = 2,465) were annotated as *Euryarchaeota* and 9% (*n* = 265) were affiliated to the Marine Group II. We further focused on functional genes reported earlier in the literature: genes involved in motility, energy pump, defense, and light harvesting (Tables S3 and S4).

**Figure 6:**
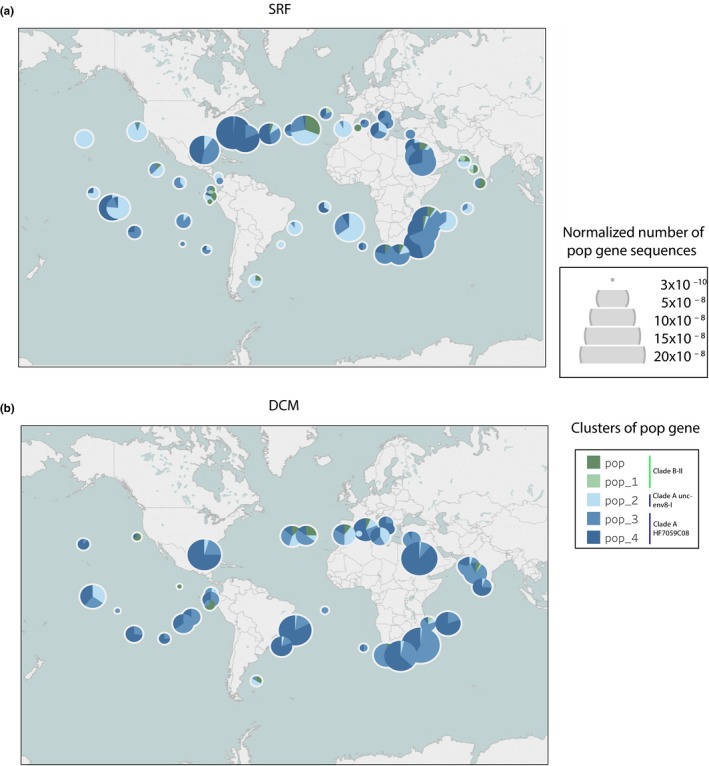
Map showing the relative proportion of the different proteorhodopsin gene clusters in the SRF (a) and DCM (b) *Tara* Oceans samples

Sequences coding for proteorhodopsin were associated to clusters 23, 27, and 3, and the gene associated to cluster 27 was from *Euryarchaeota* (>95% similarity) (Table S4). Among the *Euryarchaeota* proteorhodopsin genes (*pop*), we identified variants affiliated to pop, pop1, pop2, pop3, and pop4 according to the classification by (Iverson et al., 2012) (Figure 6). The amino acid in position 105 is indicative of the spectral tuning of the proteorhodopsin (Man et al., 2003). Our alignment showed a Methionin (M) in position 105 in the pop and pop1 variants suggestive of green light absorption (Figure S8). Pop2, pop3, and pop4 had a Glutamine (Q) at position 105 that suggests blue light absorption. All pop gene variants had amino acid residues at position 97 and 108 that demonstrate proton pumping ability (Gushchin et al., 2013; Jung, 2007) (Figure S8).

Among the *Tara* Oceans samples, euryarchaeal pop genes were detected mainly in the SRF and the DCM layers and they belonged principally to four pop groups: pop4, pop3, pop 2 and pop1 (Figure 6 and Figure S9a). Overall, there were twice as many pop and pop1 sequences in SRF compared to DCM waters (6.5% vs. 3.6%, Figure S9b). Pop2 was also more abundant in SRF compared to DCM waters (23.6% vs. 8.7%). The distribution of the different pop clades was not homogenous across the globe. In SRF waters, some samples were dominated by pop2, other by pop3 and some by pop4 (Figure 6). In deep waters, some samples were dominated by pop3 and other by pop4. There was no correlation between the abundance of pop variants in SRF versus DCM samples (Figure S10).

Other functional genes had a MGII affiliation. For example, cluster 2 was associated to six different genes coding for mastigonemes (hairs covering the flagella) that were annotated as MGII (82%–99% similarity), two genes coding for 4Fe–4S (94.5%–96% similarity), and two genes coding for TraB/PrgY‐like protein (metalloprotease) (96%–97.9%). Genes coding for luciferase co‐occurred with the cluster number 2, 27, 25, and one of these genes was annotated as MGII and another as *Euryarchaeota* (blast similarity up to 94%). Genes coding for sodium and proton pump co‐occurred with clusters 2, 3, 4, 12, 15, 23, 27, and some were from *Euryarchaeota* (up to 97.5% similarity) (Tables S3 and S4). Two genes involved in drug resistance were associated to the cluster 2 (transporters and drug permeases) and affiliated as *Euryarchaeota*. In addition, sequences coding for a catalase/peroxidase were associated to cluster 3, 4 with a *Euryarchaeota* affiliation (92% similarity) (Tables S3 and S4).

### 3.6 Gene conservation between the ribosomal operon genomic regions of uncultivated marine group II contigs

**Figure 7:**
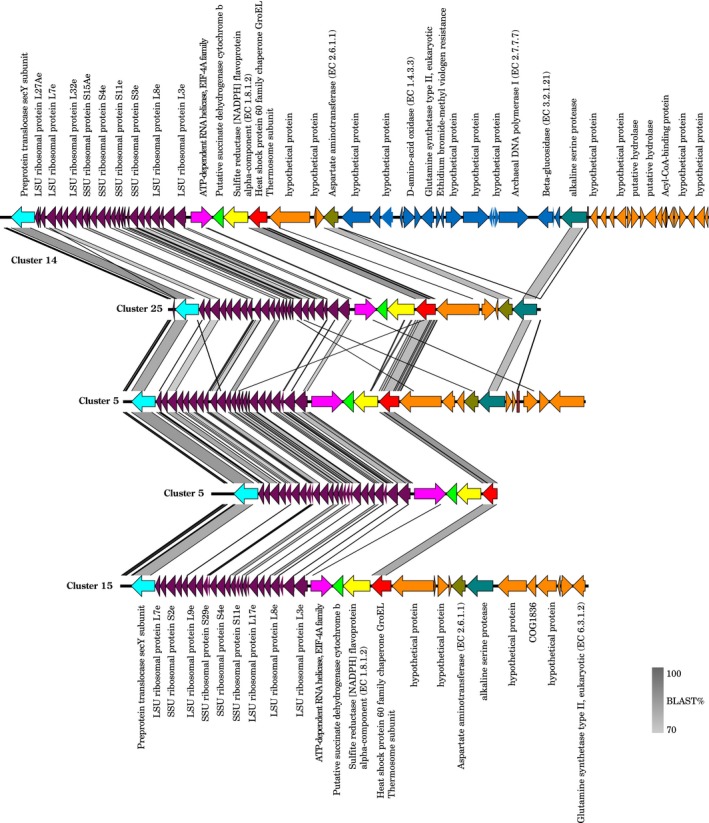
Visualization of gene annotation and gene conservation between five MGII contigs. Arrows represent genes and colors represent conserved genes. The shades of gray are proportional to the percentage of similarity between sequences

A total of 450 contigs were affiliated to MGII (Table S5) and we further analyzed the largest contigs (a total of 5) (Table S6). The five largest 16S rRNA containing contigs with a MGII affiliation were identified as representatives for the clusters 14, 25, 5, and 15. Overall, the five contigs had a similar structure with the exception of the cluster 14 representative that had a set of additional genes (Figure 7). All contigs had the ribosomal operon positioned at the same location, between a highly conserved preprotein translocase *sec*Y subunit and a less conserved ATP dependent RNA helicase of the EIF‐4A family. The helicase was always followed by a poorly conserved (blastp <70%) putative succinate dehydrogenase cytochrome b subunit, a sulfite reductase NADPH flavoprotein alpha component, and a well conserved heat shock protein 60 family chaperone GroEL/ thermosome subunit (Figure 7). The orientation of the genes was always the same. The two contigs corresponding to clusters 5 had a conserved ribosomal operon, but the intergenic regions were less conserved. The contigs of clusters 14, 25, 5, and 15 had an aspartate aminotransferase and an alkaline serine protease. The aspartate aminotransferase was conserved between cluster 14 and 25 (blastp >70%) and the alkaline serine protease between clusters 14, 25, 5 (blastp >70%). In addition, cluster 14 had a hypothetical protein and a structural protein (COG1836), a D‐amino‐acid oxidase, a putative nudix hydrolase, a glutamine synthetase type II with eukaryotic origin, an ethidium bromide‐ methyl viologen resistance protein EmrE, an archaeal DNA pol I, and a betaglucosidase. This set of genes was inserted between the ATP dependent RNA helicase and the alkaline deshydrogenase.

## 4 DISCUSSION

Our metagenome based study shows that marine archaea were ubiquitous and that the relative proportion of archaeal reads increased with depth across the eight major oceanic regions sampled by *Tara* Oceans. This global report of the distribution of marine archaea revises the pioneering work by Massana et al. (2000). At the time, they analyzed eight samples covering different oceanic region and showed by clone libraries the presence of cosmopolitan archaeal phylotypes (Massana et al., 2000). An increasing archaeal abundance with depth has also been observed earlier but these were local observations in the Pacific Ocean (Karner, DeLong, & Karl, 2001; Lincoln et al., 2014; Massana, Murray, Preston, & DeLong, 1997; Pernthaler, Pernthaler, & Amann, 2002), or the North Atlantic (Teira, Lebaron, Aken, & Herndl, 2006) and they were based on PCR or probe based methods with their inherent bias. Furthermore, we show that globally *Euryarchaeota* was the phylum that dominated in the upper layers of the ocean, while *Thaumarchaeota* were systematically more abundant with depth. Interestingly, we could also identify representatives of less common phyla such as the *Bathyarchaeota* or the newly described *Woesearchaeota* phylum present in some deep‐sea samples from the India Ocean and from North Pacific Ocean in large proportions (up to 50%). The important presence of these putative anerobic microorganisms with a fermentative and/or symbiosis‐based lifestyles (Castelle et al., 2015) in planktonic samples is surprising but could be associated to the presence of particles or sediments in the samples, or to a new marine aerobic lineage.

The use of co‐occurrence networks allowed us to move further in the direction of a functional description of the distribution of archaea by defining genomic environments. A genomic environment could be defined as the set of genes found together with a specific microorganism. These genes can be from the microorganism's own genome but they can also belong to other co‐occurring microorganisms. All these genes would define the potential metabolisms that are present, the interactions between organisms and the interactions with the environment. The genomic environment could thus theoretically characterize the organisms and the ecological niche that it occupies. The characterization of a genomic environment of uncultured organisms would allow to define ecologically meaningful units in complement to the ecotype model of genetically and ecologically distinct units (Cohan, 2006). We identify several ecological Marine Group II units characterized by a specific surrounding environment and set of associated genes. These genes were either shared with other clusters in the network or unique to a cluster.

In the euphotic zone, cluster 27 co‐occurred with a large number of genes and among which one for a proteorhodopsin identified earlier in a MGII clone (Frigaard et al., 2006), which could give beneficial supplemental energy to the archaeal cell (Iverson et al., 2012). In the global ocean, the MGII proteorhodopsin genes were present in both the SRF and the DCM layers but their distribution was not homogenous (Figure 6). The proteorhodopsin detected here belonged mainly to pop2, 3, and 4, as annotated by Iverson et al. (2012), and affiliated to the rhodopsin Clade A. They represent the medium GC clade and the high GC clades shown in Frigaard et al. (2006). The 16S rRNA of *Euryarchaeota* containing the pop4 proteorhodopsin variant belong to a clade diverging from the MGIIa according to Iverson et al. (2012). Our analysis of the pop genes amino acid composition showed that they had proton pumping ability and that pop2, 3, and 4 absorbed light in the blue range, which is the color typically found in deeper waters. An absorption spectra adaptation is known for bacteria proteorhodopsin (Béjà, Spudich, Spudich, Leclerc, & DeLong, 2001). There are different pigment family variants with absorption maxima spanning from blue (490 nm) to green (540 nm) (Béjà et al., 2001). The color tuning is thought to represent adaptations to depth or coastal‐open ocean transitions (Pinhassi et al., 2016). The green tuned pop and pop1 genes of the rhodopsin clade B (Iverson et al., 2012) were more present in surface waters, but they were not abundant. It indicates that these variants, although detected earlier (Iverson et al., 2012; Martin‐Cuadrado et al., 2015; Rinke et al., 2018), are not the most common in the global ocean. These light spectra are more common in surface waters but the pop and pop1 gene variants have been found in both surface (Iverson et al., 2012) and DCM layers (Martin‐Cuadrado et al., 2015; Rinke et al., 2018). Our results confirm that the different pop variants tend to vary geographically, and less vertically.

Cluster 3, which was relatively more abundant in the surface and DCM, co‐occurred with a catalase gene previously seen on MGII contigs (Tully, Sachdeva, Graham, & Heidelberg, 2017; Xie et al., 2018). The presence of a catalase gene suggests that some MGII have to deal with oxidative stress associated with phototrophs that produce reactive oxygen species (Xie et al., 2018). In surface waters, we also detected genes for beta‐glucosidase associated to the cluster 14, which was associated to higher water temperatures. Beta‐glucosidase is involved in the extra cellular cleavage of polysaccharide often present as high molecular weight dissolved organic matter. Beta‐glucosidase activity is stimulated by phytoplankton blooms (Arrieta & Herndl, 2002) so we could hypothesize that some MGII, like cluster14, could be using algal produced carbon in warmer seas (Orsi et al., 2015). The cluster 14 contig that we analyzed was different from the other contigs in terms of gene composition and had a set of additional genes, although the analysis was limited to regions adjacent to the ribosomal operon (Figure 7).

In the deep layers (MES), the RDA and SIMPER analysis revealed three typical deep water MGII Euryarchaeota: cluster 15, 6, and 2. Cluster 2, which belonged to the Marine Group IIb known to contain typical deep sea archaea (Deschamps et al., 2014; Martin‐Cuadrado et al., 2008; Massana et al., 2000; Moreira et al., 2004), was overall the cluster that had the highest number of co‐occurring genes earlier annotated as MGII (17.4%). Cluster 15 and 6 had the largest number of shared MGII genes. Many of the genes were not characterized or were coding core functions. Cluster 2 co‐occurred with genes involved in assimilatory sulfate reduction and MGII genes coding for surface adhesion. The potential for adhesion could suggest a lifestyle associated to particles. Particles and MGII interactions have been observed earlier in the euphotic zone of the central California Current System (Orsi et al., 2015). The particle attached life style could be associated with an anerobic metabolism as suggested by the detection of genes possibly involved in nitrate reductase (Rinke et al., 2018). In addition, several mesopelagic clusters co‐occurred with genes possibly involved in the extracellular degradation of proteins and fatty acids. Our observations thus extend earlier local observation to the world's ocean and suggest a global implication of some MGII taxa in the anerobic degradation of marine particles.

We also detected sequence coding for molybdopterin oxidoreductase, a 4Fe–4S ferredoxin (iron‐clustering protein) and a TraB/PrgY‐like metalloprotease protein (Table S4), which could be indication for the anerobic respiration of dimethylsulphoxide (DMSO) (Martin‐Cuadrado et al., 2008). Finally, we noted that cluster 2 co‐occurred with an ammonia oxidation gene (*amoA)* affiliated to *Thaumarchaeota*. This result supports recent findings demonstrating a co‐occurrence of some MGIIb and some *Thaumarchaeota* OTUs (Parada & Fuhrman, 2017).

On a methodological note, we would like to emphasize that our study provides a detailed assessment of the global distribution of planktonic MGII archaea without PCR or probe based approaches. PCR or probes are essential tools in microbial ecology, which have revealed the diversity and distribution of marine archaea (Galand et al., 2009; Hugoni et al., 2013; Karner et al., 2001; Massana et al., 2000; Pernthaler et al., 2002; Teira et al., 2006), but they have known biases (Acinas, Sarma‐Rupavtarm, Klepac‐Ceraj, & Polz, 2005; Pinto & Raskin, 2012). The metagenome 16S rRNA approach that we used, also called miTAG (Logares et al., 2014), overcomes PCR biases and may give a more realistic picture of the distribution of marine microorganisms. Our co‐occurence approach proved itself to be very efficient in associating genes to archaeal phylotypes. One indication of the power of the approach is that out of the thousands of gene coding sequence analyzed, of which a majority are bacterial, a large proportion of the ones that had a strong correlation to our archaeal clusters had been earlier annotated as archaeal.

## CONFLICT OF INTERESTS

The authors declare that they have no conflict of interest.

## AUTHOR CONTRIBUTIONS

O.P., C.H., J.C.A., D.D., and P.E.G. conceived and designed experiments and contributed to the writing of the manuscript. O.P. conducted data analysis.

## ETHICS STATEMENT

None required.

## DATA ACCESSIBILITY

The supplementary file containing sequences used for the K‐mer analysis and the Tables S1ȓS10 have been deposited to Figshare: https://doi.org/10.6084/m9.figshare.7964420.v2.
