# Supplementary figures and images for: Genomic ecology of Marine Group II, the most common marine planktonic Archaea across the surface ocean
Source: Microbiologyopen. 2019 Jul 2;8(9):e00852. doi: 10.1002/mbo3.852 (PMC6741140; doi:10.1002/mbo3.852)

## Database construction

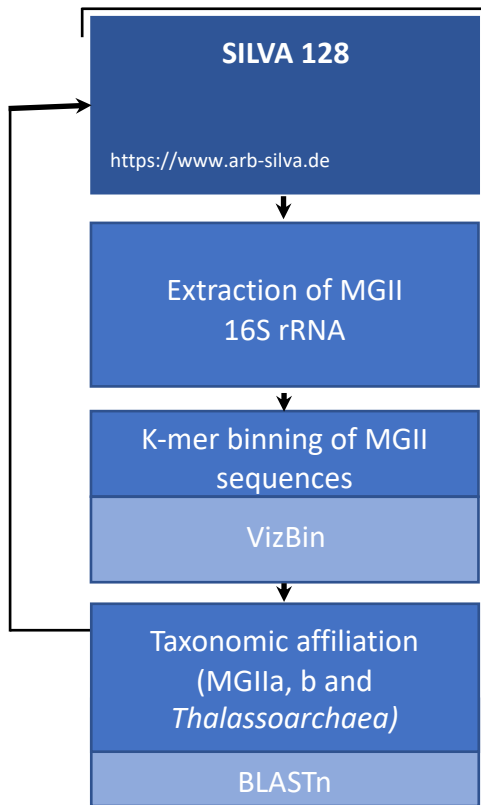

## Taxonomic analyses

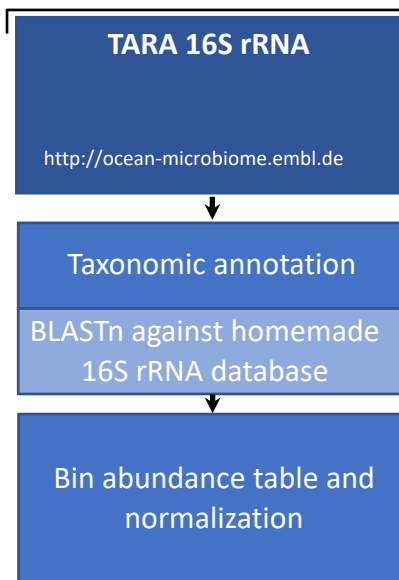

## Functional analyses

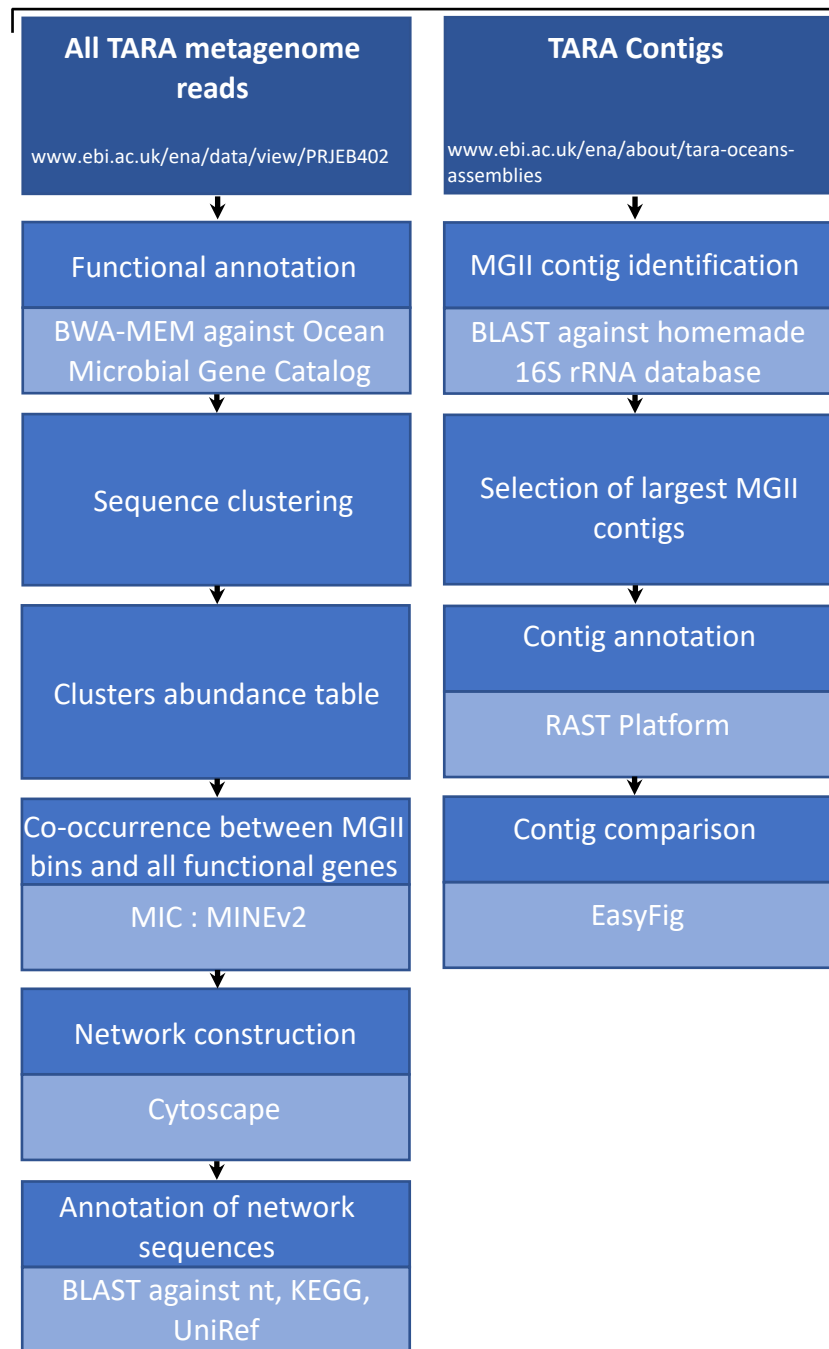

Supplement: Supplementary file 1 [file MBO3-8-e00852-s001.pdf]

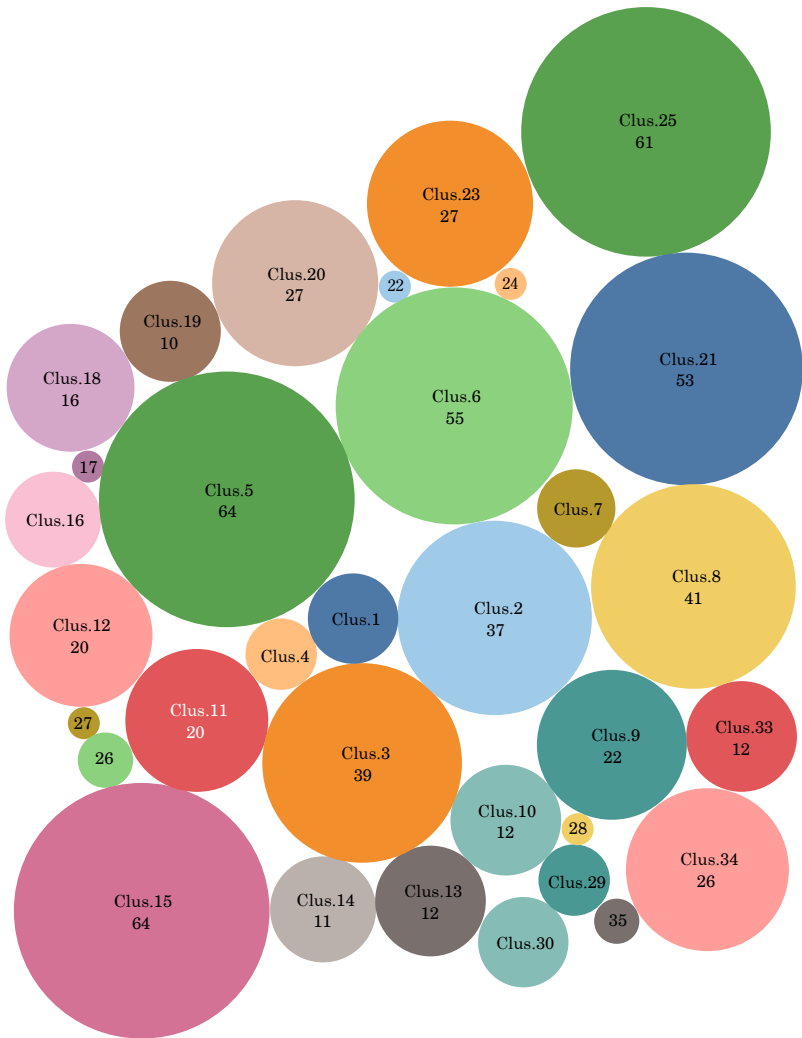

Supplement: Supplementary file 3 [file MBO3-8-e00852-s003.pdf]

## BIOSYNTHESIS OF AMINO ACIDS

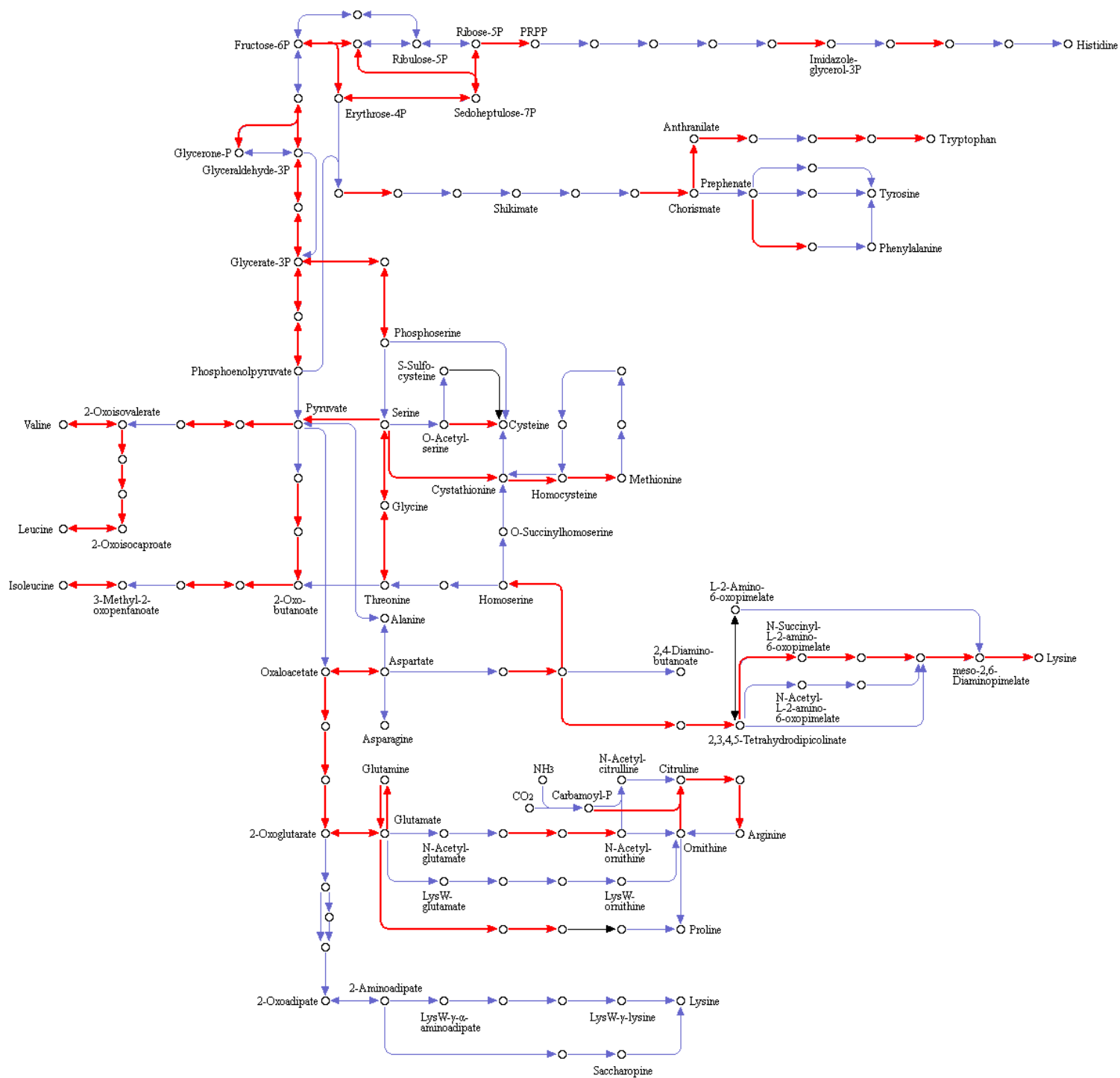

Supplement: Supplementary file 6 [file MBO3-8-e00852-s006.pdf]

## FATTY ACID DEGRADATION

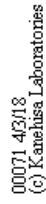

## FATTY ACID DEGRADATION

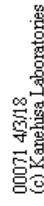

Supplement: Supplementary file 7 [file MBO3-8-e00852-s007.pdf]

MES

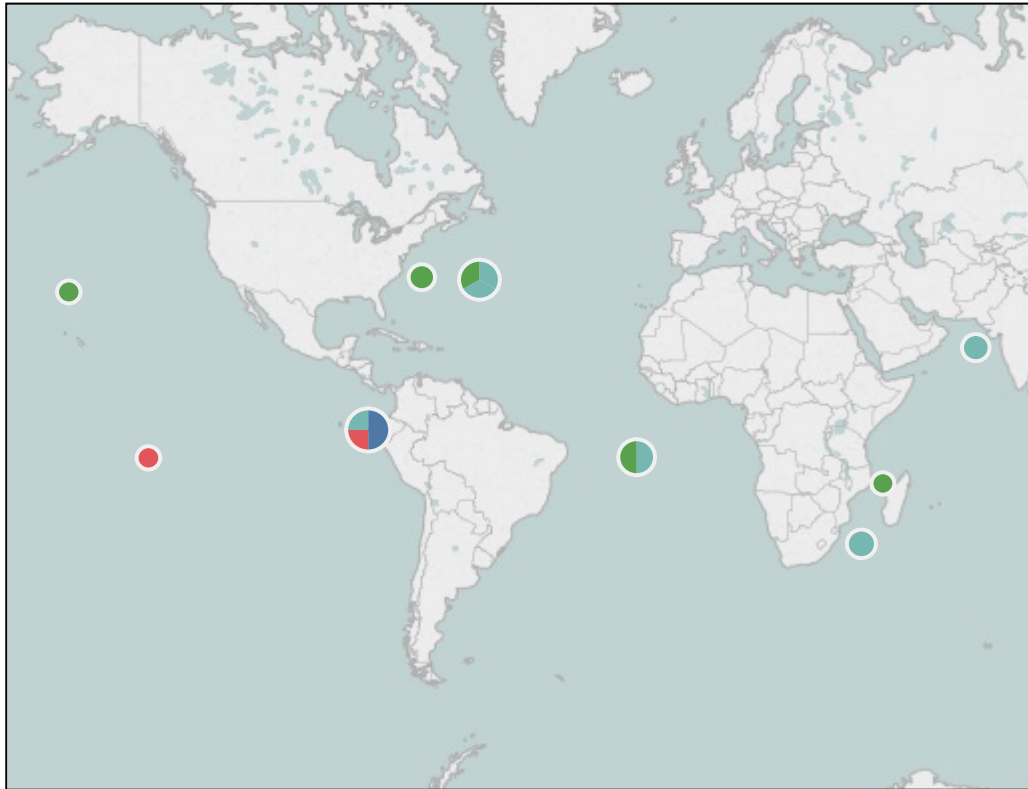

Normalized number of pop gene sequences

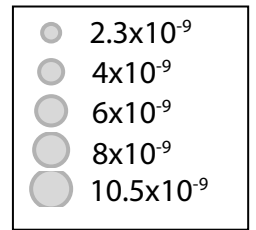

Clusters of pop gene

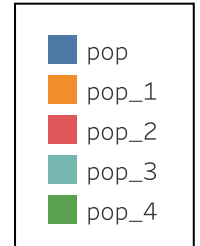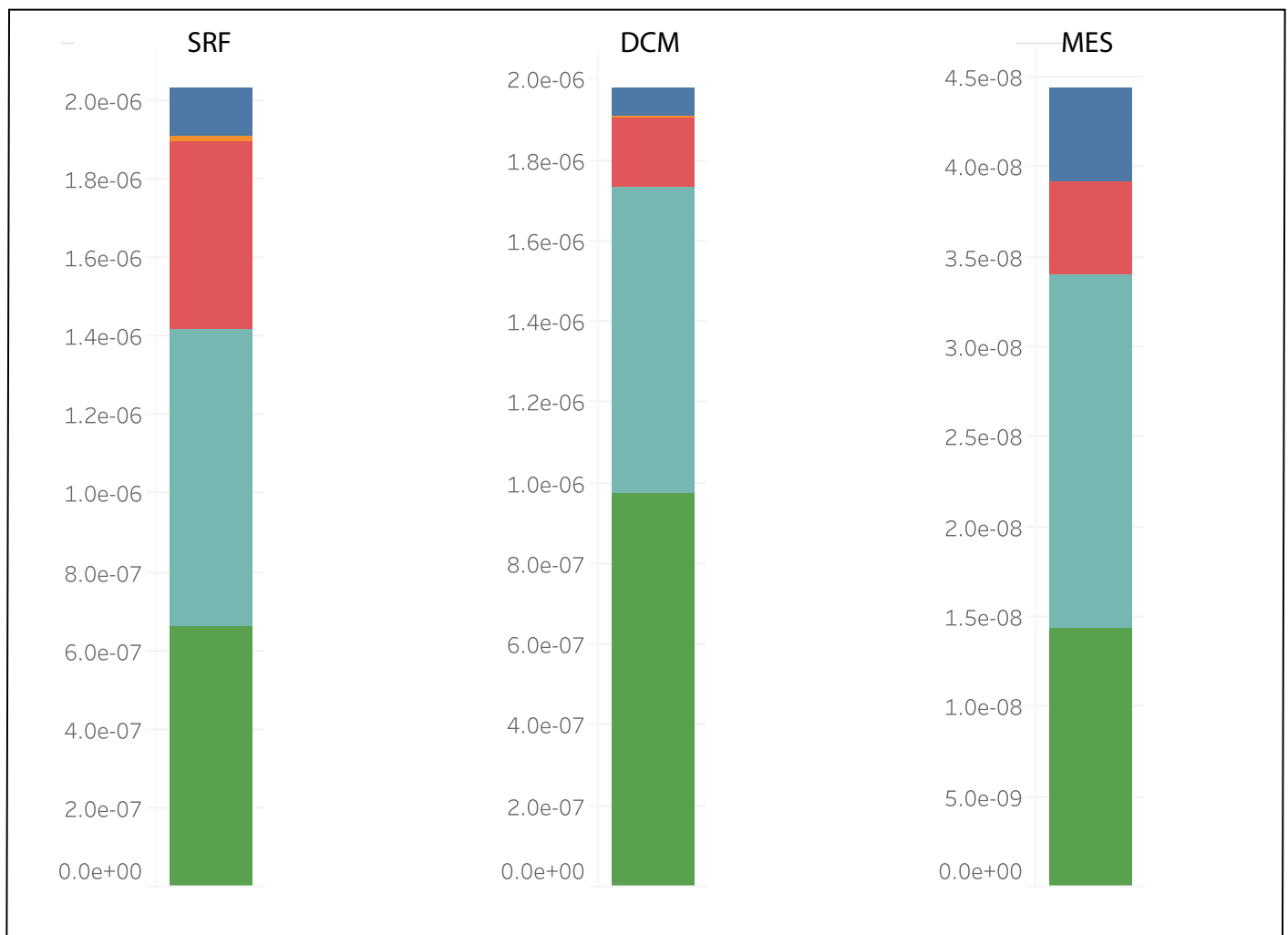

Supplement: Supplementary file 9 [file MBO3-8-e00852-s009.pdf]

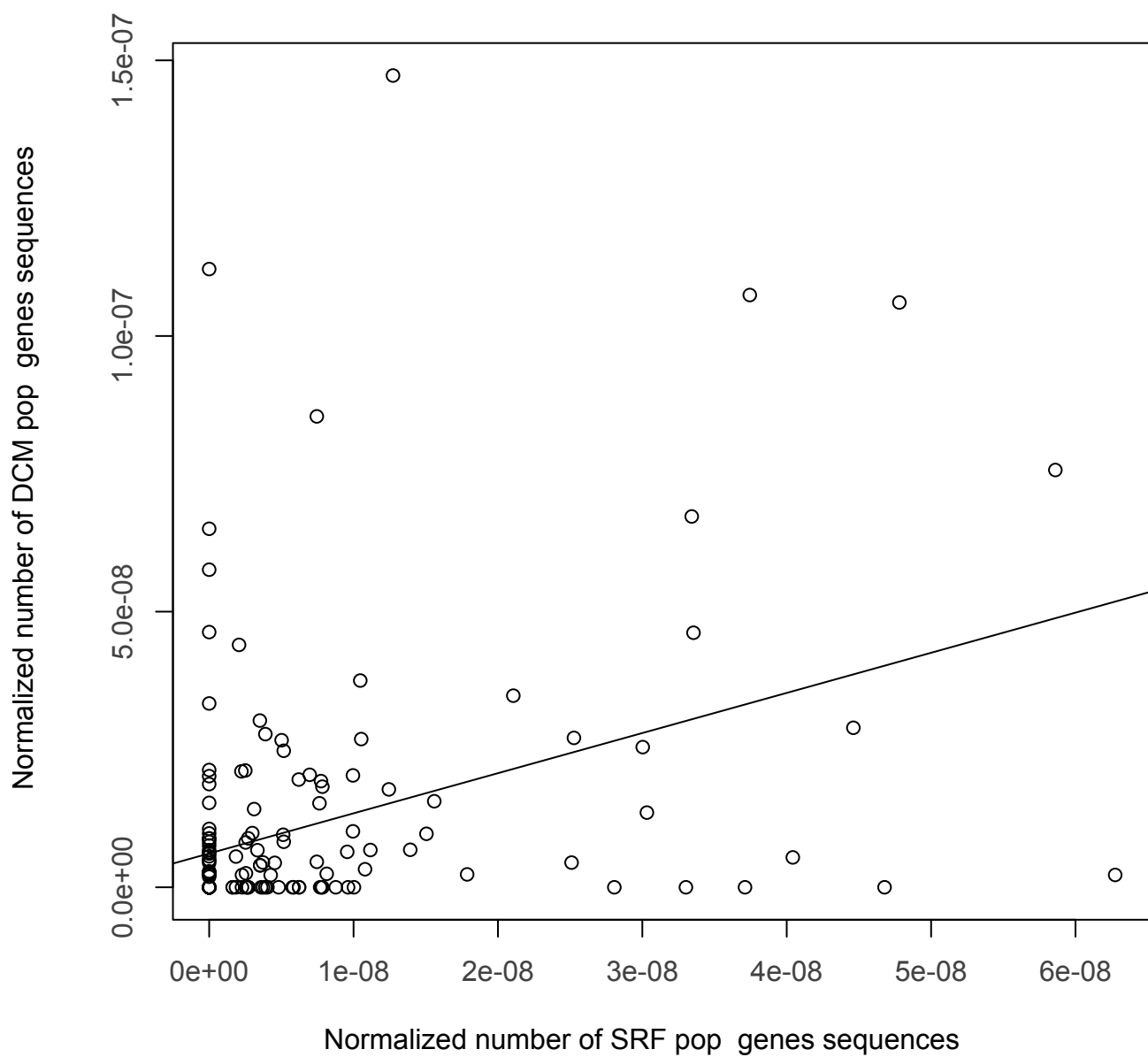

Supplement: Supplementary file 10 [file MBO3-8-e00852-s010.pdf]
