# Supplementary material for: Genomic ecology of Marine Group II, the most common marine planktonic Archaea across the surface ocean
Source: Microbiologyopen. 2019 Jul 2;8(9):e00852. doi: 10.1002/mbo3.852 (PMC6741140; doi:10.1002/mbo3.852)
Supplement: Supplementary file 2 [file MBO3-8-e00852-s002.pdf]

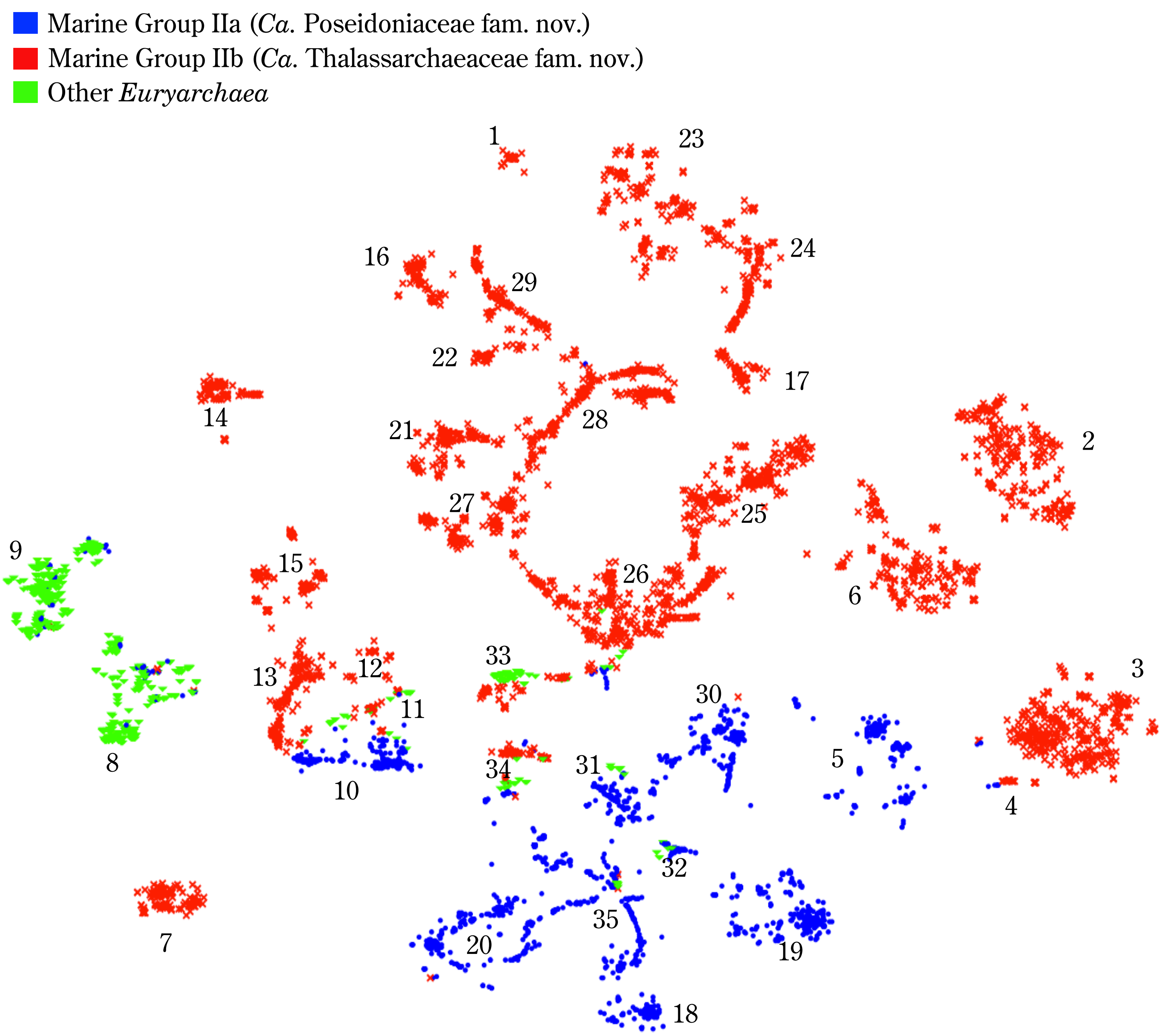
Marine Group IIa (*Ca.* Poseidoniaceae fam. nov.)
Marine Group IIb (*Ca.* Thalassarchaeaceae fam. nov.)
Other *Euryarchaea*
1
2
3
4
5
6
7
8
9
10
11
12
13
14
15
16
17
18
19
20
21
22
23
24
25
26
27
28
29
30
31
32
33
34
35
