# Supplementary material for: Genomic ecology of Marine Group II, the most common marine planktonic Archaea across the surface ocean
Source: Microbiologyopen. 2019 Jul 2;8(9):e00852. doi: 10.1002/mbo3.852 (PMC6741140; doi:10.1002/mbo3.852)
Supplement: Supplementary file 5 [file MBO3-8-e00852-s005.pdf]

(a)

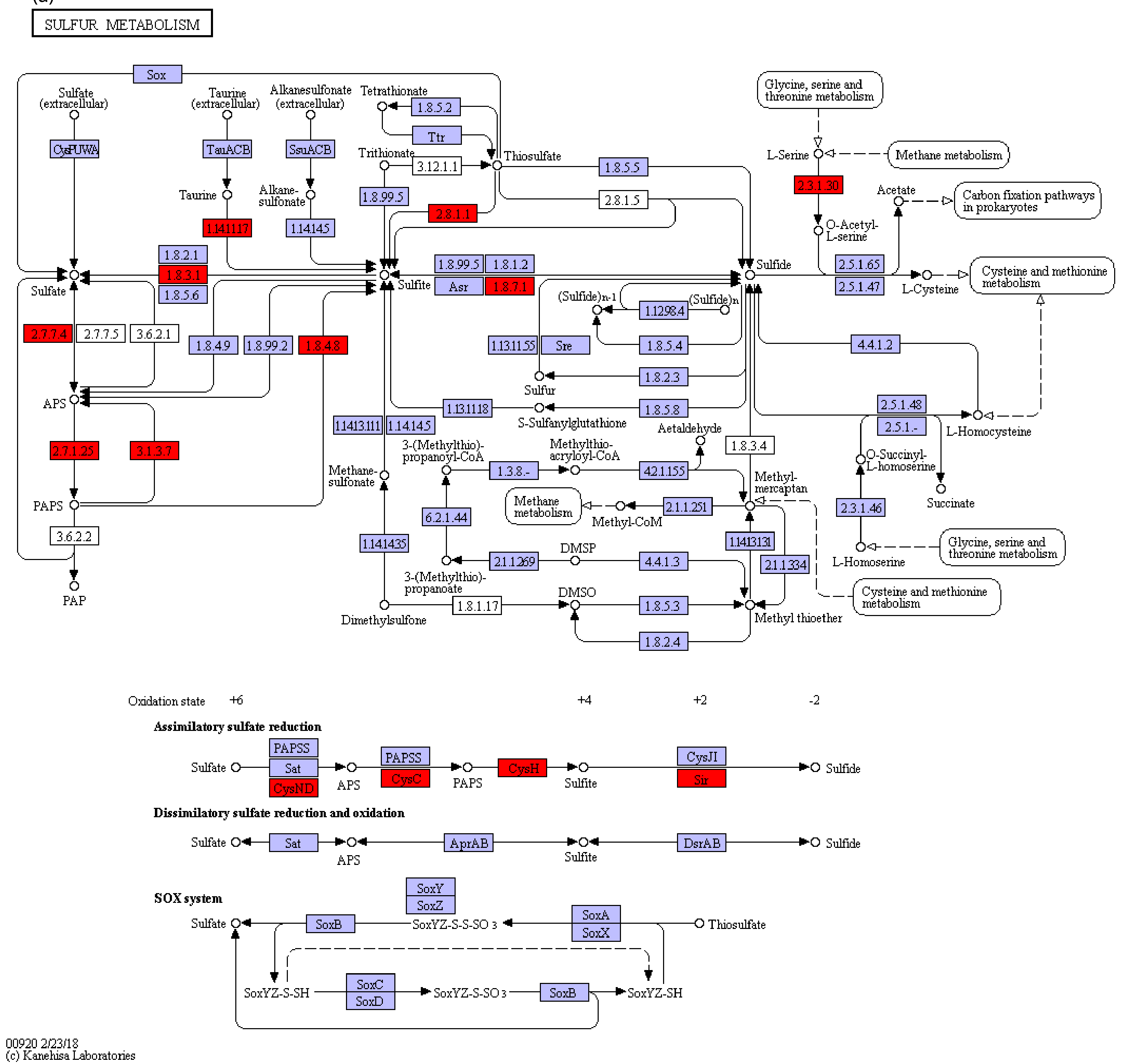
SULFUR METABOLISM
Sox
Sulfate (extracellular)
CysPUWA
Taurine (extracellular)
TauACB
Alkanesulfonate (extracellular)
SsuACB
Tetrathionate
1.8.5.2
Ttr
Trithionate
3.12.1.1
Thiosulfate
1.8.5.5
2.8.1.5
Taurine
1.14.11.17
Alkane-sulfonate
1.14.14.5
1.8.99.5
2.8.1.1
1.8.2.1
1.8.3.1
1.8.5.6
Sulfate
Sulfite
1.8.99.5
1.8.1.2
Asr
1.8.7.1
Sulfide
2.7.7.4
2.7.7.5
3.6.2.1
1.8.4.9
1.8.99.2
1.8.4.8
APS
2.7.1.25
3.1.3.7
PAPS
3.6.2.2
PAP
(Sulfide)n-1
1.12.98.4
(Sulfide)n
1.8.5.4
1.13.11.55
Sre
1.8.2.3
Sulfur
1.13.11.18
1.8.5.8
S-Sulfanylglutathione
1.14.13.111
1.14.14.5
Glycine, serine and threonine metabolism
L-Serine
Methane metabolism
2.3.1.30
Acetate
Carbon fixation pathways in prokaryotes
O-Acetyl-L-serine
2.5.1.65
2.5.1.47
L-Cysteine
Cysteine and methionine metabolism
4.4.1.2
2.5.1.48
2.5.1.-
L-Homocysteine
O-Succinyl-L-homoserine
Succinate
2.3.1.46
L-Homoserine
Glycine, serine and threonine metabolism
Cysteine and methionine metabolism
Aetaldehyde
1.8.3.4
3-(Methylthio)-propanoyl-CoA
1.3.8.-
Methylthio-acryloyl-CoA
4.2.1.155
Methyl-mercaptan
Methane-sulfonate
Methane metabolism
Methyl-CoM
2.1.1.251
6.2.1.44
1.14.13.131
1.14.14.35
2.1.1.269
DMSP
4.4.1.3
2.1.1.334
3-(Methylthio)-propanoate
Dimethylsulfone
1.8.1.17
DMSO
1.8.5.3
Methyl thioether
1.8.2.4
Oxidation state
+6
+4
+2
-2
Assimilatory sulfate reduction
Sulfate
PAPSS
Sat
CysND
APS
PAPSS
CysC
PAPS
CysH
Sulfite
CysJI
Sir
Sulfide
Dissimilatory sulfate reduction and oxidation
Sulfate
Sat
APS
AprAB
Sulfite
DsrAB
Sulfide
SOX system
Sulfate
SoxB
SoxY
SoxZ
SoxYZ-S-S-SO3
SoxA
SoxX
Thiosulfate
SoxYZ-S-SH
SoxC
SoxD
SoxYZ-S-SO3
SoxB
SoxYZ-SH
00920 2/23/18
(c) Kanehisa Laboratories

(b)

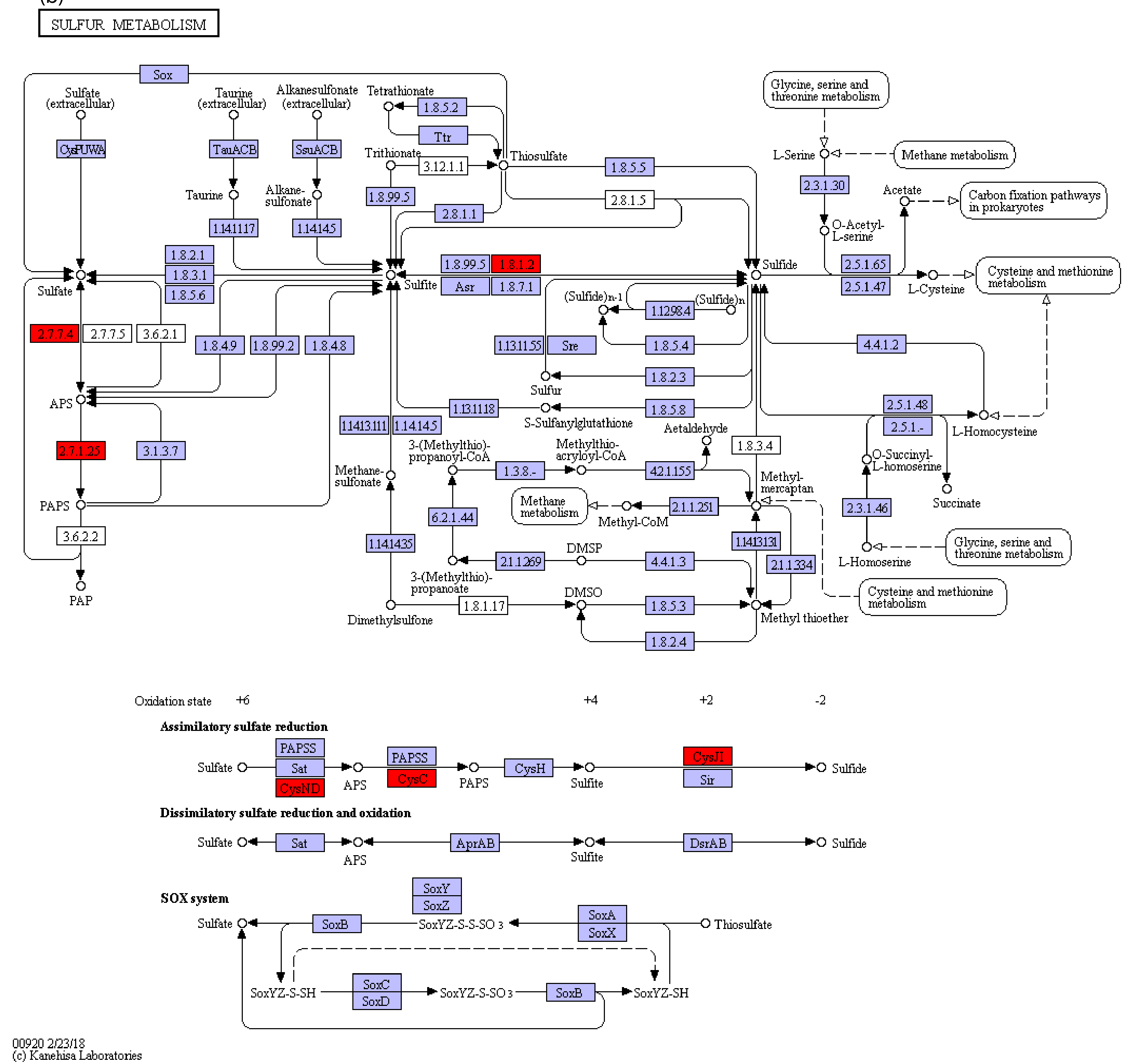
SULFUR METABOLISM
Sox
Sulfate (extracellular)
CysPUWA
Taurine (extracellular)
TauACB
Alkanesulfonate (extracellular)
SsuACB
Tetrathionate
Trithionate
Thiosulfate
Taurine
Alkane-sulfonate
1.14.11.17
1.14.14.5
1.8.5.2
Ttr
3.12.1.1
1.8.5.5
2.8.1.5
1.8.99.5
2.8.1.1
Sulfate
Sulfite
Sulfide
1.8.2.1
1.8.3.1
1.8.5.6
1.8.99.5
1.8.1.2
Asr
1.8.7.1
2.7.7.4
2.7.7.5
3.6.2.1
1.8.4.9
1.8.99.2
1.8.4.8
APS
2.7.1.25
3.1.3.7
PAPS
3.6.2.2
PAP
(Sulfide)n-1
(Sulfide)n
1.12.98.4
1.13.11.55
Sre
1.8.5.4
1.8.2.3
Sulfur
1.13.11.18
1.8.5.8
S-Sulfanylglutathione
1.14.13.111
1.14.14.5
Methane-sulfonate
1.14.14.35
3-(Methylthio)-propanoyl-CoA
1.3.8.-
Methylthio-acryloyl-CoA
4.2.1.155
Aetaldehyde
1.8.3.4
Methyl-mercaptan
6.2.1.44
Methane metabolism
Methyl-CoM
2.1.1.251
1.14.13.131
DMSP
2.1.1.269
4.4.1.3
2.1.1.334
3-(Methylthio)-propanoate
Dimethylsulfone
1.8.1.17
DMSO
1.8.5.3
Methyl thioether
1.8.2.4
Glycine, serine and threonine metabolism
L-Serine
Methane metabolism
2.3.1.30
Acetate
Carbon fixation pathways in prokaryotes
O-Acetyl-L-serine
2.5.1.65
2.5.1.47
L-Cysteine
Cysteine and methionine metabolism
4.4.1.2
2.5.1.48
2.5.1.-
L-Homocysteine
O-Succinyl-L-homoserine
Succinate
2.3.1.46
L-Homoserine
Glycine, serine and threonine metabolism
Cysteine and methionine metabolism
Oxidation state +6 +4 +2 -2
Assimilatory sulfate reduction
Sulfate
PAPSS
Sat
CysND
APS
PAPSS
CysC
PAPS
CysH
Sulfite
CysJI
Sir
Sulfide
Dissimilatory sulfate reduction and oxidation
Sulfate
Sat
APS
AprAB
Sulfite
DsrAB
Sulfide
SOX system
Sulfate
SoxB
SoxY
SoxZ
SoxYZ-S-S-SO3
SoxA
SoxX
Thiosulfate
SoxYZ-S-SH
SoxC
SoxD
SoxYZ-S-SO3
SoxB
SoxYZ-SH
00920 2/23/18
(c) Kanehisa Laboratories

(c)

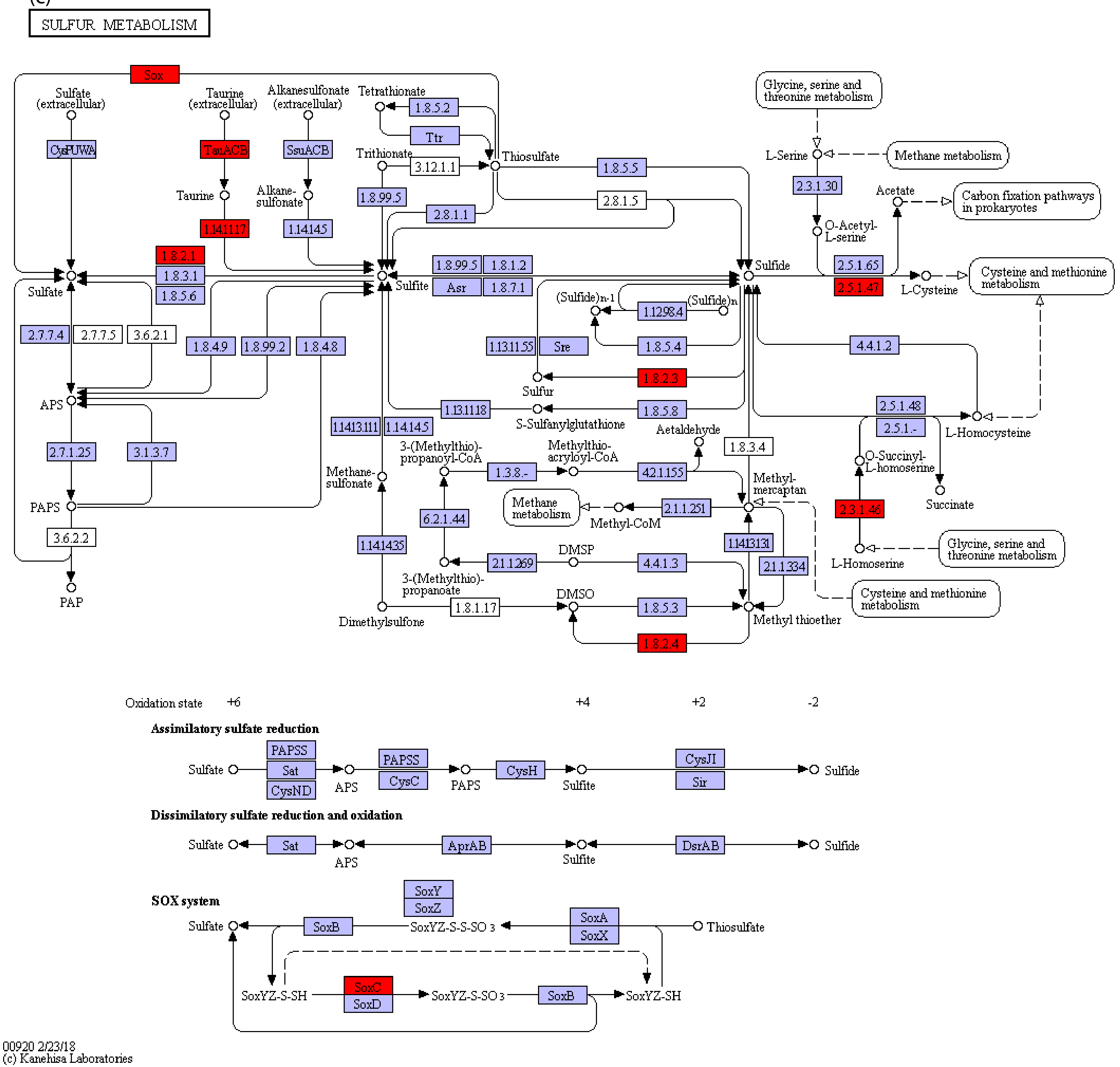
SULFUR METABOLISM
Sox
Sulfate (extracellular)
CysPUWA
Taurine (extracellular)
TauACB
Taurine
1.14.11.17
Alkanesulfonate (extracellular)
SsuACB
Alkane-sulfonate
1.14.14.5
Tetrathionate
1.8.5.2
Ttr
Trithionate
3.12.1.1
Thiosulfate
1.8.5.5
2.8.1.5
1.8.99.5
2.8.1.1
Sulfate
1.8.2.1
1.8.3.1
1.8.5.6
2.7.7.4
2.7.7.5
3.6.2.1
1.8.4.9
1.8.99.2
1.8.4.8
APS
2.7.1.25
3.1.3.7
PAPS
3.6.2.2
PAP
Sulfite
1.8.99.5
1.8.1.2
Asr
1.8.7.1
Sulfide
(Sulfide)n-1
(Sulfide)n
1.12.98.4
1.13.11.55
Sre
1.8.5.4
1.8.2.3
Sulfur
1.13.11.18
1.8.5.8
S-Sulfanylglutathione
1.14.13.111
1.14.14.5
Methane-sulfonate
1.14.14.35
3-(Methylthio)-propanoyl-CoA
1.3.8.-
Methylthio-acryloyl-CoA
4.2.1.155
Aetaldehyde
1.8.3.4
6.2.1.44
Methane metabolism
Methyl-CoM
2.1.1.251
Methyl-mercaptan
1.14.13.131
3-(Methylthio)-propanoate
2.1.1.269
DMSP
4.4.1.3
2.1.1.334
Dimethylsulfone
1.8.1.17
DMSO
1.8.5.3
Methyl thioether
1.8.2.4
Glycine, serine and threonine metabolism
L-Serine
Methane metabolism
2.3.1.30
O-Acetyl-L-serine
Acetate
Carbon fixation pathways in prokaryotes
2.5.1.65
2.5.1.47
L-Cysteine
Cysteine and methionine metabolism
4.4.1.2
2.5.1.48
2.5.1.-
L-Homocysteine
O-Succinyl-L-homoserine
Succinate
2.3.1.46
L-Homoserine
Glycine, serine and threonine metabolism
Cysteine and methionine metabolism
Oxidation state +6 +4 +2 -2
Assimilatory sulfate reduction
Sulfate
PAPSS
Sat
CysND
APS
PAPSS
CysC
PAPS
CysH
Sulfite
CysJI
Sir
Sulfide
Dissimilatory sulfate reduction and oxidation
Sulfate
Sat
APS
AprAB
Sulfite
DsrAB
Sulfide
SOX system
Sulfate
SoxB
SoxY
SoxZ
SoxYZ-S-S-SO3
SoxA
SoxX
Thiosulfate
SoxYZ-S-SH
SoxC
SoxD
SoxYZ-S-SO3
SoxB
SoxYZ-SH
00920 2/23/18
(c) Kanehisa Laboratories

(d)

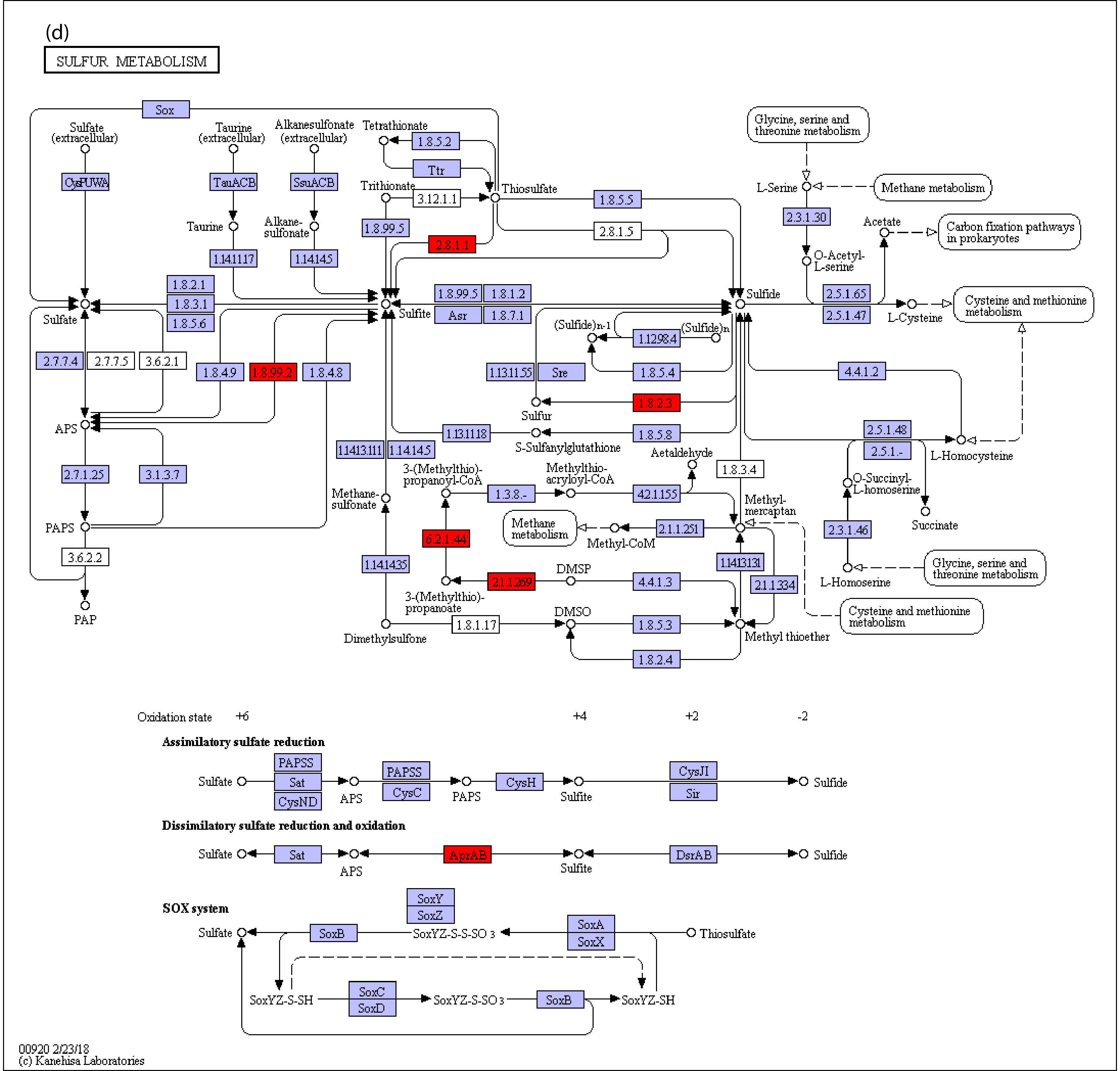

SULFUR METABOLISM
Sox
Sulfate (extracellular)
CysPUWA
Taurine (extracellular)
TauACB
Alkanesulfonate (extracellular)
SsuACB
Tetrathionate
1.8.5.2
Ttr
Trithionate
3.12.1.1
Thiosulfate
1.8.5.5
2.8.1.5
1.8.99.5
2.8.1.1
Taurine
Alkane-sulfonate
1.14.11.17
1.14.14.5
1.8.2.1
1.8.3.1
1.8.5.6
Sulfate
Sulfite
1.8.99.5
1.8.1.2
Asr
1.8.7.1
Sulfide
2.7.7.4
2.7.7.5
3.6.2.1
1.8.4.9
1.8.99.2
1.8.4.8
APS
2.7.1.25
3.1.3.7
PAPS
3.6.2.2
PAP
(Sulfide)n-1
(Sulfide)n
1.12.98.4
1.13.11.55
Sre
1.8.5.4
1.8.2.3
Sulfur
1.13.11.18
1.8.5.8
S-Sulfanylglutathione
1.14.13.111
1.14.14.5
Methane-sulfonate
3-(Methylthio)-propanoyl-CoA
1.3.8.-
Methylthio-acryloyl-CoA
4.2.1.155
Aetaldehyde
1.8.3.4
Methyl-mercaptan
Methane metabolism
Methyl-CoM
2.1.1.251
6.2.1.44
1.14.14.35
1.14.13.131
2.1.1.269
DMSP
4.4.1.3
2.1.1.334
3-(Methylthio)-propanoate
Dimethylsulfone
1.8.1.17
DMSO
1.8.5.3
1.8.2.4
Methyl thioether
Glycine, serine and threonine metabolism
L-Serine
Methane metabolism
2.3.1.30
Acetate
Carbon fixation pathways in prokaryotes
O-Acetyl-L-serine
2.5.1.65
2.5.1.47
L-Cysteine
Cysteine and methionine metabolism
4.4.1.2
2.5.1.48
2.5.1.-
L-Homocysteine
O-Succinyl-L-homoserine
Succinate
2.3.1.46
L-Homoserine
Glycine, serine and threonine metabolism
Cysteine and methionine metabolism
Oxidation state
+6
+4
+2
-2
Assimilatory sulfate reduction
Sulfate
PAPSS
Sat
CysND
APS
PAPSS
CysC
PAPS
CysH
Sulfite
CysJI
Sir
Sulfide
Dissimilatory sulfate reduction and oxidation
Sulfate
Sat
APS
AprAB
Sulfite
DsrAB
Sulfide
SOX system
Sulfate
SoxB
SoxY
SoxZ
SoxYZ-S-S-SO3
SoxA
SoxX
Thiosulfate
SoxYZ-S-SH
SoxC
SoxD
SoxYZ-S-SO3
SoxB
SoxYZ-SH
00920 2/23/18
(c) Kanehisa Laboratories
