# Supplementary material for: Genomic ecology of Marine Group II, the most common marine planktonic Archaea across the surface ocean
Source: Microbiologyopen. 2019 Jul 2;8(9):e00852. doi: 10.1002/mbo3.852 (PMC6741140; doi:10.1002/mbo3.852)
Supplement: Supplementary file 8 [file MBO3-8-e00852-s008.pdf]

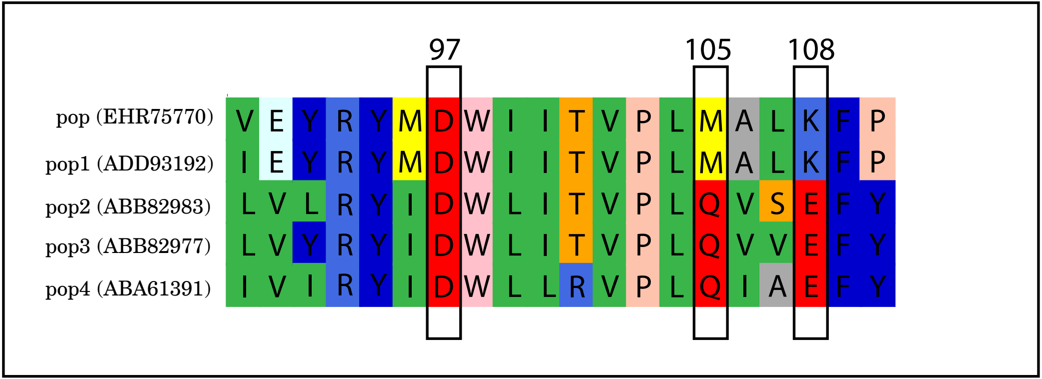
97
105
108
pop (EHR75770) V E Y R Y M D W I I T V P L M A L K F P
pop1 (ADD93192) I E Y R Y M D W I I T V P L M A L K F P
pop2 (ABB82983) L V L R Y I D W L I T V P L Q V S E F Y
pop3 (ABB82977) L V Y R Y I D W L I T V P L Q V V E F Y
pop4 (ABA61391) I V I R Y I D W L L R V P L Q I A E F Y
